# Superior results of return to sport after double-bundle versus single-bundle anterior cruciate ligament reconstruction in young active patients

**DOI:** 10.1007/s00167-022-07010-6

**Published:** 2022-06-02

**Authors:** Liang Qin, Hongbo You, Jun Qi, Ye Ren, Peng Cheng, Shuang Liang, Jiang Wang

**Affiliations:** Department of Orthopedics, Tongji Hospital, Tongji Medical College, Huazhong University of Science and Technology, 1095 Jie Fang Avenue, Wuhan, 430030 People’s Republic of China

**Keywords:** Anterior cruciate ligament reconstruction, Anterior cruciate ligament, Double-bundle technique, Single-bundle technique, Return to sport

## Abstract

**Purpose:**

To compare return to sport and clinical results in young active patients who underwent anatomic single-bundle (SB) versus double-bundle (DB) anterior cruciate ligament reconstruction (ACLR).

**Methods:**

Young active patients undergoing SB or DB ACLR from 2017 to 2019 at our institution were retrospectively reviewed. The primary outcome measures were the rate and time to return to sports, with secondary measures including the Lachman test, pivot shift test, Lysholm scores, International Knee Documentation Committee (IKDC) scores and graft rupture.

**Results:**

The study included a total of 90 patients (DB group, 42; SB group, 48), with a mean follow-up of 27.1 ± 6.1 months. Young active patients who underwent DB ACLR had a higher rate of return to pivoting sports than those who underwent SB ACLR (HR = 2.4; 95% confidence interval [CI]: 1.4, 4.1; *p* = 0.013). The DB group returned to pivoting sports at a mean ± SD of 11.0 ± 2.9 months compared with 12.7 ± 2.7 months in the SB group (*p* = 0.01). There was one traumatic failure in the SB group and one contralateral ACL rupture in the DB group. There was no significant difference in the rate and time to return to running, Lachman test, pivot-shift test, Lysholm or IKDC scores in either group.

**Conclusion:**

Both anatomical SB and DB techniques achieved satisfactory clinical outcomes. DB techniques led to superior performance of return to pivoting sports but nonsignificant differences in time and rate of return to running, passive stability measurement, subjective knee function outcome and graft rupture rate in both groups at the 2-year follow-up. The DB ACLR should be considered a viable option to treat young patients with high activity demands.

**Level of evidence:**

III.

## Introduction

Return to sports and the capacity to resume sporting activities are among the main expectations of young active patients undergoing ACL surgery. Although ACL reconstruction (ACLR) has successfully improved clinical results in many patients with ACL rupture, only 70% were able to return to their preinjury participation levels in young patients [2]. Furthermore, a high incidence of reinjury to the ipsilateral or contralateral knee has been reported, particularly in younger patients who return to sports following ACLR [6, 21]. These data suggest that there is still a need for improvement of current treatment protocols and reconstruction techniques in young active patients.

To overcome unresolved problems after ACLR, advanced techniques, including anatomic single-bundle (SB) and double-bundle (DB) reconstruction, have been developed in the past several decades. Many clinical and biomechanical studies have compared clinical outcomes and knee kinematics between anatomic SB and DB ACLR, and the results remain controversial [3, 7, 10, 12, 20]. It is unclear whether these previous findings include data from patients of all age ranges and activity levels lead to the results. As the demands of different activity levels or age range populations lead to different study outcomes [11, 18], there is a strong need for sport-specific or age-specific clinical outcome studies between DB and SB ACLR.

The primary objective of this study was to compare the return to sports, knee stability, functional results and reinjury rate between the 2 ACL reconstruction techniques in young active patients at the 2-year follow-up. The primary hypothesis underlying this study was that the DB ACL reconstruction method would demonstrate superior performance of return to sport in young active patients. Second, it was hypothesised that the DB ACL reconstruction method would result in a lower graft rupture rate, better knee stability, and better subjective findings than the SB method in young active patients. These results may have implications for decisions regarding selecting the method of ACL reconstruction when individualising treatment in young active patients with high activity demands.

## Materials and methods

IRB approval (TJ-A202170401) from Tongji Hospital affiliated to Tongji Medical College of Huazhong University of Science and Technology was granted for this study. In accordance with the inclusion and exclusion criteria, patients who underwent ACL reconstruction in our institute were retrospectively reviewed between 2017 and 2019. Briefly, patients were eligible to participate in the study if they (1) had an ACL-deficient knee requiring; (2) were skeletally mature (3) were no more than 25 years of age at the time of surgery; and (4) participated in a professional or competitive level I pivoting sport. Patients were excluded if they (1) had undergone previous ACL reconstruction on either knee; (2) required bilateral ACL reconstruction; (3) required surgical repair or reconstruction of the posterior cruciate ligament, medial collateral ligament, lateral collateral ligament, or posterolateral corner; or (4) had a symptomatic articular cartilage defect requiring treatment other than debridement. Before each patient was admitted for surgery, the patient was allowed to select the method of ACL reconstruction after the benefits and the risks were described and informed consent was acquired.

One hundred and five young active patients underwent the ACLR procedure from 2017 to 2019 at Tongji Hospital affiliated to Tongji Medical College of Huazhong University of Science and Technology, of whom 90 patients (85.7%)(42 in the DB group and 48 in the SB group) were available for final follow-up. The mean follow-up time was 27.1 ± 6.1 months (range, 19–44 months) after surgery. There was no difference between the two groups with regard to age, sex, or left and right knees. There were 51 patients with meniscus injuries, for whom the menisci were sutured, shaped, or resected according to the type of injury. One patient in the DB group tore their contralateral ACL and required ACLR operation. One patient in the SB group had a traumatic failure for instability during the follow-up and went on to undergo revision ACL surgery. Therefore, the statistical analysis concerning the return to sport and clinical results was conducted using data from 88 patients (41 in the DB group and 47 in the SB group). The demographic and clinical characteristics of each group are shown in Table 1.

**Table 1:** Patient demographics

Parameter	DB group	SB group	*p* Value
No. of patients	42	48	N/A
Average age at surgery (years)	22.0 ± 2.5	22.3 ± 2.3	n.s.
Male sex	31 (73.8)	34 (70.8)	n.s.
Injury side, right/left	29/13	32/16	n.s.
BMI (kg/m^2^)	22.4 ± 2.7	22.3 ± 2.3	n.s.
Time from injury to surgery (months)	3.2 ± 1.9	2.9 ± 1.8	n.s.
Meniscal lesions, *n* (%)	28	23	n.s.
Months of follow-up	26.6 ± 5.8	27.5 ± 6.3	n.s.
Cause of injury, *n* (%)			n.s.
Sport	35 (83.3)	42 (87.5)	
Traffic accident	5 (11.9)	4 (8.3)	
Work	2 (4.8)	2 (4.2)	
Preoperative level of sport, *n* (%)			n.s.
Professional	8 (19.0)	8 (16.7)	
Competitive	34 (81.0)	40 (83.3)	
Recreational	0 (0)	0 (0)	

Values are presented as mean ± SD or *n* (%); BMI, body mass index;SB, single-bundle reconstruction; DB, double-bundle reconstruction; N/A, not applicable

One senior surgeon performed all the reconstructions. A standard arthroscopic examination was performed through the anteromedial and anterolateral portals. A ruptured ACL was confirmed arthroscopically, and meniscal injury was managed according to the injury status. Serving as grafts, the hamstring tendon tendons were exposed and harvested. For SB ACLR, both tendons were used as a 4-strand graft, with a total diameter of 8–10 mm; For DB reconstruction, the semitendinosus tendons were used as a double-strand graft to replace the AM bundles, with a diameter of 7–8 mm, and the double-strand gracilis tendons were used as the PL bundles, with a diameter of 6–7 mm. Tibial fixation was performed with aperture fixation and suspensory fixation was used on the femur.

The postoperative rehabilitation protocol was the same for all patients. Based upon the examination, jogging and running were usually permitted at 3 months after surgery, and return to sport activities involving jumping, pivoting, or side-stepping was allowed at 9 months postoperatively when quadriceps power reached 90% compared with the contralateral leg.

For each patient, demographics, cause of injury, preoperative level of sport, preoperative stability measurement outcomes(Lachman test, and a manual pivot-shift test), preoperative knee function scores (International Knee Documentation Committee and Lysholm knee scores) and surgery information were collected as part of the routine intake assessment.

Information on return to sport was collected by interviewing records at the following postoperative time periods: 1, 6, 12, 18, 24 months and last follow-up. Patients were asked whether they returned to running, how many months after surgery they had restarted running, whether they returned to pivoting sports, and how many months after surgery they had restarted pivoting sports.

The evaluation methods consisted of a clinical examination, which included stability measurements using a Lachman test and a manual pivot-shift test. In addition, knee function was assessed by the International Knee Documentation Committee (IKDC) and Lysholm knee scores preoperatively and at the last follow-up. Graft rupture was also evaluated with MRI at the last follow-up. All clinical assessments were performed by one independent examiner.

### Statistical analysis

Statistical analysis was performed using the SPSS 11.0 software package (SPSS Inc., Chicago, IL, USA). The results are expressed using mean values ± standard deviation (SD) for parametric values. Statistical comparison between the preoperative and follow-up parametric scores was performed using paired Student's *t*-test, while the Mann–Whitney *U* test was used to compare parametric variables between the SB and DB subgroups. The chi-square test was used for categorical data. For the primary end point (return to pivoting sports and return to running in months), Kaplan–Meier curves with cumulative survival curves in the two groups were plotted. To determine the treatment effect of intervention on the rate to return to pivoting sports, a Cox proportional hazards model was performed to compute hazard ratios (HR). A *p *value < 0.05 was considered statistically significant. Sample size was determined using the free program StatBox (https://www.biostats.cn/statbox/) for power analysis. The minimum sample size required based on previous similar studies was found to be 102. In this study, 105 patients were included.

## Results

**Fig. 1:**
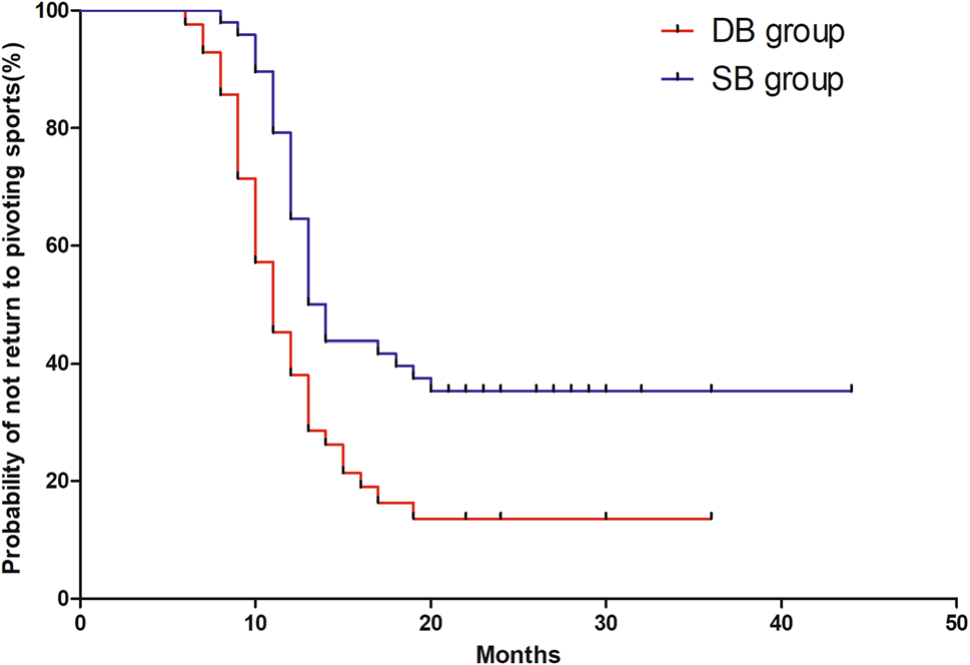
Kaplan–Meier curves showing the cumulative incidence of return to pivoting sport (SB, single-bundle reconstruction; DB, double-bundle reconstruction)

**Fig. 2:**
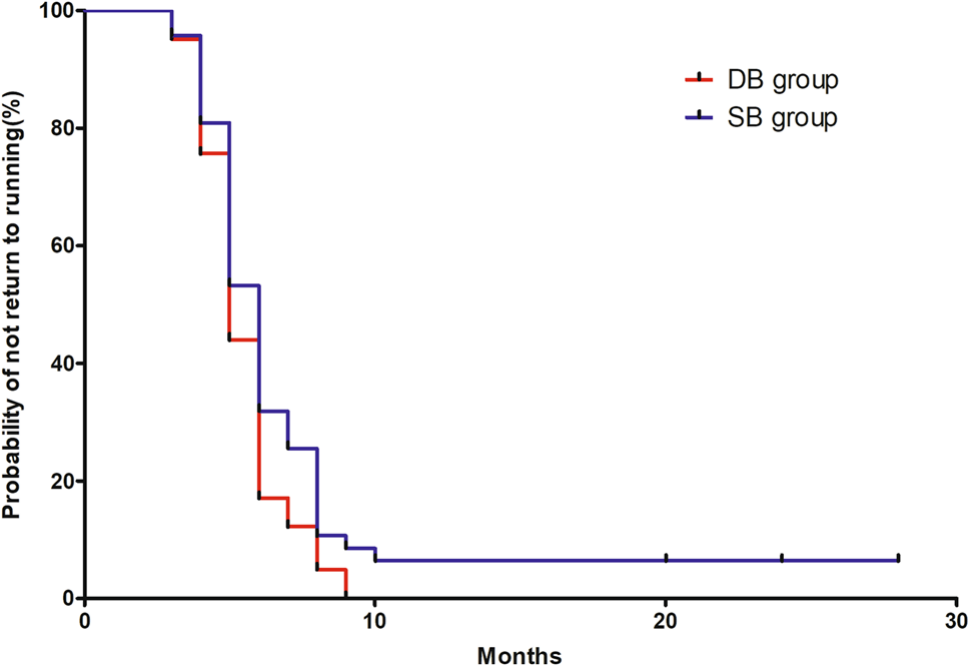
Kaplan–Meier curves showing the cumulative incidence of return to running (SB, single-bundle reconstruction; DB, double-bundle reconstruction)

In total, 96.6% and 76.1% of young active patients were able to return to running exercise and pivoting sports after successful reconstruction at the last follow-up. The time taken to return to the running exercise and return to pivoting sport following ACLR are shown in Table 2. A Kaplan–Meier survival curve that compares the probability of not returning to pivoting sports in the DB group with those in the SB group is provided in Fig. 1. Young active patients who underwent DB ACLR had a higher rate of return to pivoting sports than those who underwent SB ACLR (HR = 2.41; 95% confidence interval [CI]: 1.41, 4.10; *p* = 0.013).This HR may be interpreted as meaning that at any given point in time, a patient who underwent DB ACLR has a 2.41 times greater chance of return to pivoting sports compared with a patient who underwent SB ACLR. The Cox regression model did not identify significant differences in time to return to running (HR = 1.63; 95% confidence interval [CI]: 0.94, 2.80; n.s.) (Fig. 2).

**Table 2:** Time of return to sport

	DB group (*n* = 41)	SB group (*n* = 47)	*p* Value
Months to return running, mean ± SD	5.8 ± 1.6	5.5 ± 1.5	n.s
Months to return pivoting sports, mean ± SD	11.0 ± 2.9	12.7 ± 2.7	0.01

Values are presented as mean ± SD or *n* (%); SB, single-bundle reconstruction; DB, double-bundle reconstruction

During the follow-up period, no postoperative complications including infections, condylar fractures, restriction of the ROM or deep venous thrombosis were found in any of these patients. Comparing the two groups, significant differences regarding the Lachman test and pivot-shift test could not be seen at the final follow-up (n.s., Table 3).

**Table 3:** Objective outcomes and radiograph evaluation

	DB group (*n* = 41)	SB group (*n* = 47)	*p* Value
Pivot-shift test (overall), *n* (%)	5 (12.2)	8 (17.0)	n.s.
Normal	36	39	
Glide(+)	4	6	
Grossly positive(++)	1	2	
Lachman test, *n* (%)	8 (19.5)	12 (25.5)	n.s.
0	33	35	
1	7	10	
2	1	1	
3	0	1	

Values are presented as *n* (%); SB, single-bundle reconstruction; DB, double-bundle reconstruction.

The Lysholm and IKDC knee scores in each group were good and significantly better at the last follow-up than the corresponding values preoperatively (*p* < 0.05). However, there was no significant difference in any of these values between the groups at the last follow-up (n.s., Table 4).

**Table 4:** Subjective evaluation

	DB group (*n* = 41)	SB group (*n* = 47)	*p* Value
Lysholm score			
Preoperatively	59.3 ± 6.9	60.9 ± 7.1	n.s.
Last follow-up	93.2 ± 3.5	91.9 ± 4.0	n.s.
* p* Value	< 0.001	< 0.001	
IKDC Subjective score			
Preoperatively	57.1 ± 7.8	58.9 ± 8.1	n.s.
Last follow-up	86.7 ± 6.6	88.9 ± 6.4	n.s.
* p* Value	< 0.001	< 0.001	

Values are presented as mean ± SD;I KDC, International Knee Documentation Committee; SB, single-bundle reconstruction; DB, double-bundle reconstruction

## Discussion

The principal findings of this study of a homogenous group of young and very active patients undergoing DB or SB ACL reconstruction techniques reveal that DB techniques led to an increased rate of return to pivoting sport and decreased time to return to pivoting sport, despite there being no difference in rate and time to return to running between the two groups. There was no significant difference in the laxity measurement, subjective knee function outcome or graft rupture rate in either group at the last follow-up. Both anatomical SB and DB techniques achieved satisfactory clinical outcomes.

Recent studies have demonstrated that a range of contextual factors affect return to sport and clinical outcomes after ACLR, including age, sex, sport participation level, and psychological factors [4, 18]. Although clinical results and knee kinematics are similar in most recent studies comparing SB and DB techniques, it is unclear whether data that include ACL reconstruction in all age ranges and activity levels lead to the results. Young patients or athletes have a very intense demand for return to sports, and the level of rehabilitation exercise is relatively high. More stable and better restoring knee kinematics close to the normal ACL may lead to better sport function in young patients or athletes with high activity demands. Few authors have analysed the return to sport and clinical outcomes of DB vs. SB ACLR depending on patients’ sports activities and age. Regarding the return to sports, this study indicates that DB reconstruction is a good option and should be considered when individualising treatment in young active patients.

With increasing participation in competitive sports and the trend to start sporting activities at an early age, there has been a marked increase in the rate of ACL tears and reconstructions in young populations. Return to sport activities and especially to pivoting sport levels represents one of the most important clinical goals after ACL reconstruction for young and very active patient. Multiple case series have examined return-to-sport ability after ACLR, and rates of return to pivoting or preinjury sport vary between 40 and 83% [9, 17]. Age, sex, body mass index (BMI), preinjury sport level and preoperative quadriceps strength have been shown to be the most common factors related to these outcomes [1, 5]. In a mid- to long-term study of a group of young athletes (< 25 years old) [15], 72% of patients were able to participate in sporting activities after ACLR, but only half were able to return to their preinjury participation levels. A recent meta-analysis of 8 studies evaluating 1239 athletes younger than 20 years reported that 87% returned to sports and 80% resumed high-risk activities [1]. In studies of DB ACLR in patients under 30 years, Lim et al. [8] reported that 54.9% of patients returned to the preinjury level of sport. Among patients undergoing DB vs. SB ACLR, some previous studies [16] demonstrated no significant differences in return to sport outcome between the two techniques. Some of these observed discrepancies in return to sport outcomes can be attributed to different patient populations (age, sex, specific sport, level of participation in sports and more) and different study methodologies [13]. In this work, return to sport is defined as return to running and return to pivoting sport, respectively. Although there was no difference in return to running, our study demonstrated that DB techniques led to an increased rate of return to pivoting sport and decreased time to return to pivoting sport in young active patients. The reasons for the superior performance of return to pivoting sport in the DB group remain unclear, but we can assume that the DB grafts restore intact knee kinematics significantly better than the single-bundle technique.

Graft failure and contralateral ACL rupture can still occur even after successful ACLR. Graft failure rates after ACLR range from 3 to 25% in some populations [1]. This wide range is likely due to data that include ACL injury in all age ranges, follow-up periods, and activity levels. Previous studies have reported that younger age is a predictor for higher rates of subsequent ACL graft failure [19]. According to a report based on the Australian national registry for ACL reconstruction incidence and demographics between 2000 and 2015, the individuals at greatest risk are men aged 20–24 years and women aged 15–19 years [22]. Individual studies have cited return to high-risk sports involving jumping/landing, cutting, and pivoting as other risk factors [1]. During the follow-up period, we identified one traumatic failure in the SB group and one contralateral ACL rupture in the DB group. Several studies comparing SB and DB reconstruction found no difference in the reinjury rate [14]. Long-term evaluations in risk assessments after ACLR are important, as a significant number of subsequent ACL injuries occur later than routine follow-up.

There are, however, several limitations to the present study. First, the study was designed retrospectively, and as such, it carries inherent shortcomings. The selection of patients for SB or DB was not randomised and may have been subject to selection bias. Another limitation is that the return to sports after ACL reconstruction represents a complex outcome to evaluate. There are no standard criteria for return to sport, especially in return to “preinjury sports” or “pivot sports”, thus possibly creating a bias.

## Conclusion

There were no detectable differences in the laxity measurement, subjective knee function outcome or graft rupture rate between SB and DB ACLR at last follow-up. However, DB techniques led to an increased rate of return to pivoting sport and decreased time to return to pivoting sport, despite there being no difference in rate and time to return to running between the two groups. DB ACLR should be considered a viable option when individualising treatment in young active patients with high activity demands.
